# PD-1 inhibitors versus chemotherapy as second-line treatment for advanced esophageal squamous cell carcinoma: a meta-analysis

**DOI:** 10.1186/s12885-021-08958-3

**Published:** 2021-11-10

**Authors:** Xinxin Zhu, Qiyue Shanzhou, Danyang Li, Xuezhou Pang, Daiyuan Ma

**Affiliations:** Department of Oncology, Affiliated Hospital of North Sichuan Medical college, No.1 Maoyuan South Road, Shunqing District, Nanchong, Sichuan, 637000 China

**Keywords:** PD-1, Chemotherapy, Esophageal squamous cell carcinoma, Efficacy, Safety, meta-analysis

## Abstract

**Background:**

Aim to establish the inhibitors of programmed cell death protein 1 (PD-1) as second-line therapy for advanced esophageal squamous cell carcinoma (ESCC).

**Methods:**

Published clinical trials in the PubMed, Medline, Embase databases on PD-1 inhibitors for the treatment of ESCC were searched, along with an additional search on abstracts from the American Society of Clinical Oncology (ASCO) and European Society for Medical Oncology (ESMO) from inception to September 2021. Overall survival (OS), progression-free survival (PFS), objective response rate (ORR), disease control rate (DCR), and treatment-related adverse events (TRAEs) were synthesized using STATA.

**Results:**

A total of 1970 patients (PD-1 inhibitors: 987; chemotherapy: 983) were enrolled in five randomized controlled trials. Compared with conventional chemotherapy, second-line PD-1 inhibitors significantly improved the OS (hazard ratio [HR] = 0.73, 95% confidence interval [CI]: 0.66–0.81; *P* < 0.001) and ORR (relative risk [RR] = 1.89, 95% CI: 1.16–3.05; *P* = 0.01) of advanced ESCC patients, especially significantly prolonged the OS in the patients with positive programmed death-ligand 1 (PD-L1) status (HR = 0.64, 95% CI: 0.53–0.77; *P* < 0.001); but did not better PFS (HR = 0.88, 95% CI: 0.68–1.14; *P* = 0.330) and DCR (RR = 0.89, 95% CI: 0.59–1.37; *P* = 0.603). Moreover, PD-1 inhibitors were associated with statistically lower incidences of grade 3–5 TRAEs.

**Conclusion:**

Second line PD-1 inhibitors significantly improved the OS and ORR of patients with advanced ESCC, especially the OS of those with positive PD-L1 expression, and did not result in significant improvement in PFS and DCR. Compared to chemotherapy, second-line PD-1 inhibitors had superior safety profiles for the treatment of advanced ESCC.

## Background

Esophageal cancer (EC) is the seventh most prevalent malignancy and sixth most cause of cancer-related mortality worldwide with the highest incidence in Asia and Africa [1], where esophageal squamous cell carcinoma (ESCC) pathologically represents 90% of all EC cases [1, 2], which possesses a distinct genetic profile from that of adenocarcinoma [3, 4]. Due to the fact that more than two-thirds of EC patients with unresectable disease and more than one-half have postoperative local recurrence or metastasis within 5 years, the overall survival (OS) for advanced EC remains poor.

Fluoropyrimidine/platinum-based systemic chemotherapy remains the first-line therapeutic option for patients with advanced or metastatic ESCC, however, severe toxicities from systemic chemotherapy limit its widespread application in clinical practice. In the past several years, multiple large randomized phase III trials on various targeted treatments in EC have been published, but no promising targeted therapy drug has been identified that can improve long-term outcomes of patients with advanced ESCC [4–7]. To date, there are only a few treatment options for patients with unresectable or metastatic ESCC who progress on or are intolerant to first-line standard chemotherapy [4, 8, 9].

More recently, immune checkpoint inhibitors, represented by programmed cell death1/ligand 1 (PD-1/PD-L1) inhibitors, have made breakthroughs in cancer treatment. PD-1 inhibitors have been the focus of many clinical studies and have extensive clinical application. Because the positive expression rate of PD-L1 in ESCC reaches about 40% [10], PD-1 inhibitors are the most effective treatment candidates for EC.

In previous single-arm trials [11–13], PD-1 inhibitors have shown great anti-tumor activity in the treatment of ESCC. To date, a few randomized controlled trials (RCTs) have focused on the efficacy and safety of PD-1 inhibitors as second-line agents beyond chemotherapy for the treatment of advanced ESCC. Therefore, we conducted a meta-analysis on published data to preliminarily confirm the efficacy and safety of PD-1 inhibitors for the treatment of advanced ESCC.

## Methods

### Search strategy

A comprehensive systematic search was performed on the published literature databases of PubMed, Embase, Cochrane, and the unpublished abstract databases of the American Society of Clinical Oncology (ASCO) and European Society for Medical Oncology (ESMO) from inception to September 2021 with restrictions on English. The following terms were used: (Esophageal Neoplasms OR Esophageal Neoplasm OR Esophageal Cancer OR Esophagus Neoplasms OR Esophagus Cancer OR Esophagus Neoplasm OR Esophageal Squamous Cell Carcinoma OR ESCC) AND (nivolumab OR pembrolizumab or camrelizumab OR sintilimab OR tislelizumab OR toripalimab OR PD-1 OR programmed cell death 1) AND chemotherapy AND (randomized controlled trial OR randomized OR placebo).

### Inclusion and exclusion criteria

Studies eligible for the analyses satisfied each of the following requirements: RCTs on patients with advanced or metastatic ESCC refractory or intolerant to first-line therapy; trial participants in the PD-1 inhibitor arm received anti-PD-1 agent only, while participants in the chemotherapy arm were treated with conventional chemotherapy regimen; and the trial used at least three of the following (OS, progression-free survival [PFS], objective response rate [ORR], disease control rate [DCR], treatment-related adverse events [TRAEs] or grade 3–5 TRAEs) as outcome indicators. Exclusion criteria for all studies were as follows: studies with insufficient information from which usable data could not be extracted; and for multiple publications on the same clinical study, only the one with the most complete data was selected. Any discrepancies were resolved through group discussion.

### Extraction of data and assessment of risk of bias

Data were extracted into a standardized form for data collection, which recorded the OS and PFS, ORR, DCR, TRAEs, grade 3–5 TRAEs in each eligible study and supplementary materials, and the clinicopathological information for each study, which included the first author of the publication, the year of publication, the name of the trial, the phase of the trial, treatment line, some patients, expression status of PD-L1 (positive: tumor proportion score [TPS] ≥ 1% or combined positive score [CPS] ≥ 10, negative: TPS < 1% or CPS < 10), age, number of male patients, intervention and treatment, median follow-up. Moreover, the risk of bias was evaluated with the Cochrane Risk of Bias Tool [14]. Every trial was thoroughly processed and scored as either high, low, or unclear risk to the following criteria: random sequence generation, allocation concealment, blinding of participants and personnel, blinding of outcome assessment, incomplete outcome data, selective reporting, and other sources of bias. Two researchers independently carried out the data extraction and quality assessment. Disagreements, if any, were resolved by discussion and consensus among the two.

### Statistical analyses

All OS and PFS data from RCTs were analyzed by HR and 95% confidence interval (CI). ORR, DCR, the incidence of any grade TRAEs, and grade 3–5 TRAEs were assessed by relative risk (RR) and 95% CI. Also, OS was further stratified depending on the status of PD-L1 expression. Because the definition of positive expression of PD-L1 varied among clinical trials, we selected PD-L1 TPS ≥ 1% or CPS ≥ 10, which was assayed by immunohistochemistry staining methods, as a cutoff to identify PD-L1 positive patients in our studies. Heterogeneity among the studies was evaluated by the *I*^2^ statistic and Q test. Only the Dersimonian-Laird random-effects model was used for analysis. To explore the origin of heterogeneity, the studies were removed one-by-one by sensitivity analysis, and then new heterogeneity and new pooled effects were projected. Finally, the Egger’s or Begger’s test was executed to trace the publication bias when 10 or more studies were included, and *P* > 0.05 was considered no publication bias. All analyses were run with Stata (v 16.0) (Stata Corp, College Station, TX, USA). *P* < 0.05 was considered statistically significant.

## Results

### Search results and study characteristics

**Fig. 1:**
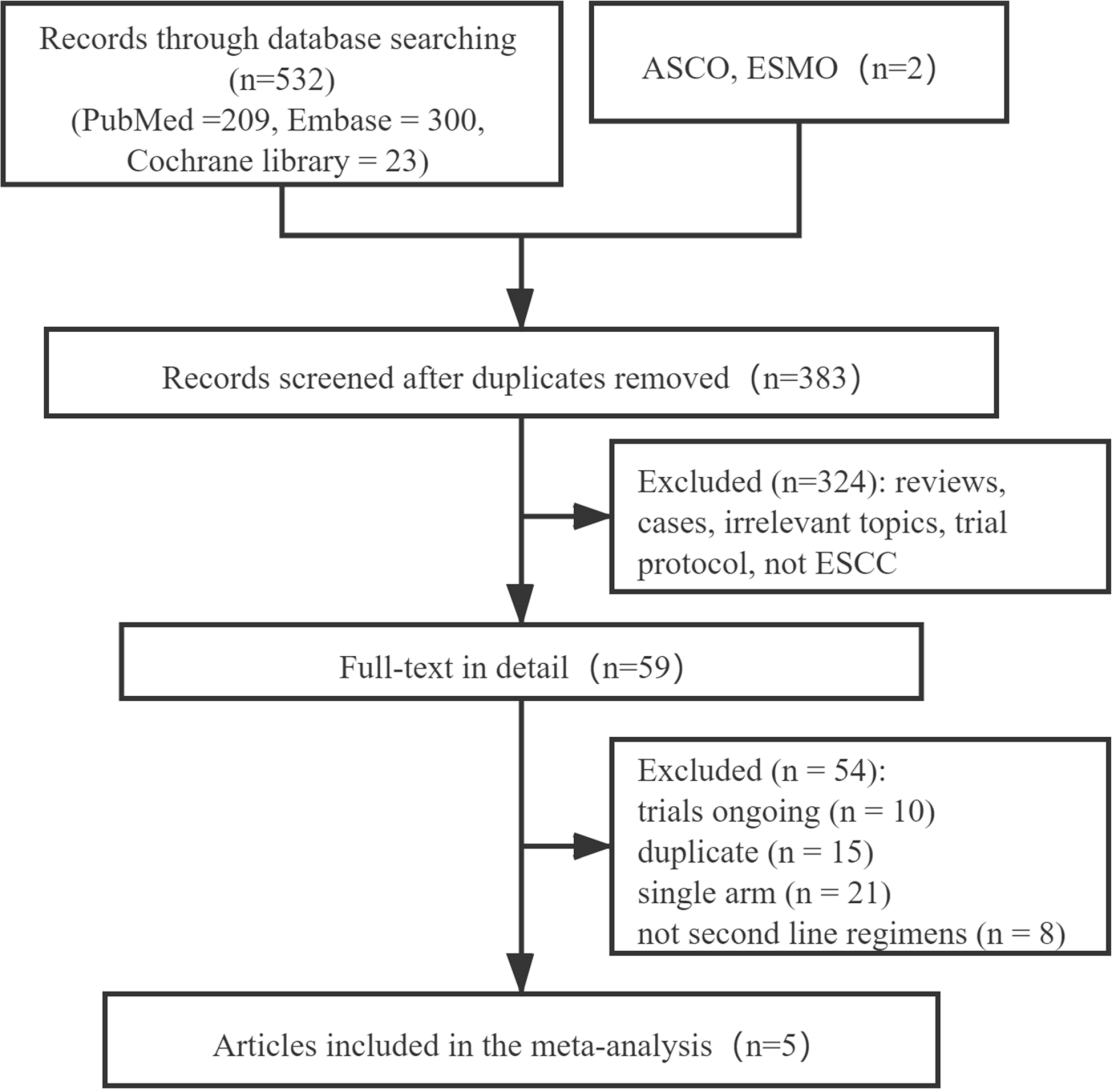
Flow diagram: selection process for the studies

By following the designed selection algorithm, 534 published or unpublished documents were retrieved, from which 151 were concealed due to duplication and 324 were disregarded just by reviewing the title and abstract. After full-text review of the remaining 59 pieces of literature, five RCTs [15–19] were selected for further analysis (Fig. 1). A total of 1970 trial participants from the five RCTs were included in this study, of which 987 received anti-PD-1 agent as a monotherapy for ESCC, while the other 983 received chemotherapy. Furthermore, three clinical trials were published from 2019 to 2020 [15–17], one of the five articles was an abstract presented at the ESMO Virtual Congress 2020 [18], and the remaining one was an abstract presented at the 2021 ASCO Virtual Annual Meeting [19]. Among the five clinical studies, three [15, 16, 19] were international multicenter phase III clinical studies and the other two [17, 18] were multicenter clinical studies from China. The immunotherapy agent was studied in each RCT as follows: pembrolizumab in KEYNOTE-181, nivolumab in ATTRACTION-3, camrelizumab in ESCORT, sintilimab in ORIENT-2 and tislelizumab in RATIONALE 302. In the KEYNOTE-181 study [15], primary endpoints were OS in patients with PD-L1 CPS ≥ 10, in patients with ESCC, and in all patients. In our study, only ESCC was included. The basic characteristics of the included literature are shown in Table 1, and the summary of outcomes was presented in Table 2.

**Table 1:** The characteristics of included studies

Study	Phase	Treatment line	Arm	Patients	PD-L1+ patients	Age, median (range)	Male (%)	ECOG (%)		Intervention
								0–1	2	
Kojima 2020 [15] KEYNOTE-181	III	2	PD-1 inhibitor	198	85	NA	NA	NA	NA	Pembrolizumab 200 mg Q3W
			Chemotherapy	203	82	NA	NA	NA	NA	Investigator choice chemotherapy
Kato 2019 [16] ATTRACTION-3	III	2	PD-1 inhibitor	210	101	64 (57, 69)	179 (85%)	210 (100%)	0 (0%)	Nivolumab 240 mg Q2W
			Chemotherapy	209	102	67 (57, 72)	185 (89%)	209 (100%)	0 (0%)	Investigator choice chemotherapy
Huang 2020 [17] ESCORT	III	2	PD-1 inhibitor	228	93	60 (54, 65)	208 (91%)	228 (100%)	0 (0%)	Camrelizumab 200 mg Q2W
			Chemotherapy	220	98	60 (54, 65)	192 (87%)	220 (100%)	0 (0%)	Investigator choice chemotherapy
Xu 2020 [18] ORIENT-2	II	2	PD-1 inhibitor	95	NA	NA	NA	95 (100%)	0 (0%)	Sintilimab 200 mg Q3W
			Chemotherapy	95	NA	NA	NA	95 (100%)	0 (0%)	Investigator choice chemotherapy
Ajani 2021 [19] RATIONALE 302	III	2	PD-1 inhibitor	256	NA	NA	NA	256 (100%)	0 (0%)	Tislelizumab 200 mg Q3W
			Chemotherapy	256	NA	NA	NA	256 (100%)	0 (0%)	Investigator choice chemotherapy

Abbreviations: NA not available, ECOG Eastern Cooperative Oncology Group performance status

**Table 2:** Summary of outcomes in the selected studies

Study	Arm	Follow-up	OS	PFS	ORR	DCR	Incidence of TRAEs		
		Median months					Any grade	Grade 3–5	Death
KEYNOTE-181	PD-1 inhibitor	7.1	8.2	2.2	16.70%	46%	64%	18.20%	1.60%
	Chemotherapy	6.9	7.1	3.1	7.40%	49.80%	86%	40.90%	1.70%
ATTRACTION-3	PD-1 inhibitor	10.5	10.9	1.7	19%	37%	65%	18%	5%
	Chemotherapy	8	8.4	3.4	22%	63%	94%	63%	4.30%
ESCORT	PD-1 inhibitor	8.3	8.3	1.9	20.20%	44.70%	94%	19%	3%
	Chemotherapy	6.2	6.2	1.9	6.40%	34.50%	90%	39%	1.40%
ORIENT-2	PD-1 inhibitor	7.2	7.2	NA	12.60%	NA	54.30%	2.02%	NA
	Chemotherapy	6.2	6.2	NA	6.30%	NA	90.80%	39%	NA
RATIONALE 302	PD-1 inhibitor	6.9	8.6	NA	20.30%	NA	NA	19%	14%
	Chemotherapy	6.9	6.3	NA	9.80%	NA	NA	56%	12%

Abbreviations: NA not available

### Study quality

All selected RCTs designated OS as the primary endpoint and were open-labeled with complete outcome data and non-selective reporting. In three RCTs [15–17], randomized treatment allocation sequences were generated with an adequate method of allocation concealment. In the remaining two RCTs [18, 19], the information regarding approaches to randomization and allocation concealment could not be determined. All five RCTs were considered to be acceptable risk of bias (Table 3).

**Table 3:** Risk of bias of RCTs

Study	Random sequence generation	Allocation concealment	Blinding of participants and personnel	Blinding of outcome assessment	Incomplete outcome data	Selective reporting	Other sources of bias
KEYNOTE-181	Low risk	Low risk	High risk	Low risk	Low risk	Low risk	Unclear risk
ATTRACTION-3	Low risk	Low risk	High risk	Low risk	Low risk	Low risk	Unclear risk
ESCORT	Low risk	Low risk	High risk	Low risk	Low risk	Low risk	Unclear risk
ORIENT-2	Unclear risk	Unclear risk	High risk	Low risk	Low risk	Low risk	Unclear risk
RATIONALE 302	Unclear risk	Unclear risk	High risk	Low risk	Low risk	Low risk	Unclear risk

### Outcome indicators

**Fig. 2:**
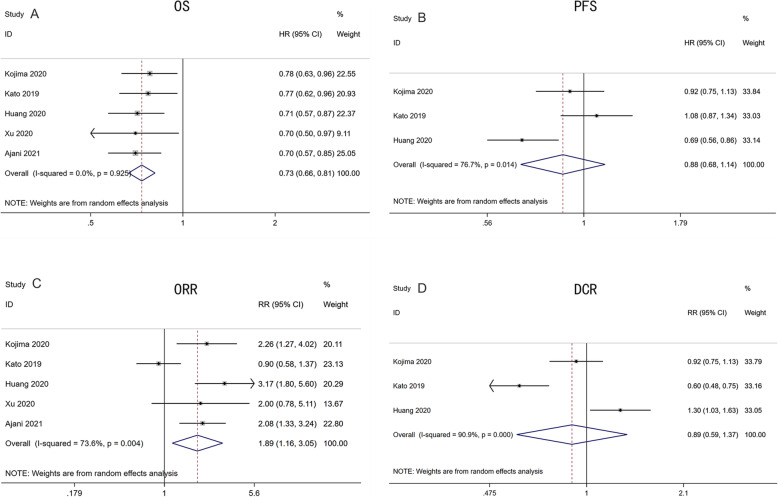
Forest plots for antitumor activity in patients treated with PD-1 inhibitors versus chemotherapy: (A) OS (B) PFS (C) ORR (D) DCR. Abbreviations. OS: overall survival; PFS: progression-free survival; ORR: objective response rate; DCR: disease control rate

OS data were available from all five studies [15–19], including 987 patients in the PD-1 inhibitor group and 983 patients in the chemotherapy group. The results showed that PD-1 inhibitors had a significantly reduced risk of death compared with chemotherapy (HR: 0.73, 95% CI: 0.66–0.81, *P* < 0.001; heterogeneity: I^2^ = 0.0%, *P* = 0.925) (Fig. 2A). PFS data were obtained from three studies [15–17] which included 636 patients in the PD-1 inhibitor group and 632 patients in the chemotherapy group, showing that PFS was not significantly favorable in the PD-1 inhibitor group (HR: 0.88, 95% CI: 0.68–1.14, *P* = 0.330; heterogeneity: I^2^ = 76.7%, *P* = 0.014) (Fig. 2B).

ORR in five studies [15–19] with 948 patients in the PD-1 inhibitor group and 932 patients in the chemotherapy group was significantly favorable in the PD-1 inhibitor group (RR: 1.89, 95% CI: 1.16–3.05, *P* = 0.01; heterogeneity: I^2^ = 73.6%, *P* = 0.004) (Fig. 2C). DCR data in three selected trials [15–17] with 597 patients in the PD-1 inhibitor arm and 581 patients in the chemotherapy arm were not significantly favorable in the PD-1 inhibitor group (RR: 0.89, 95% CI: 0.59–1.3, *P* = 0.603; heterogeneity: I^2^ = 90.9%, *P* < 0.001) (Fig. 2D).

**Fig. 3:**
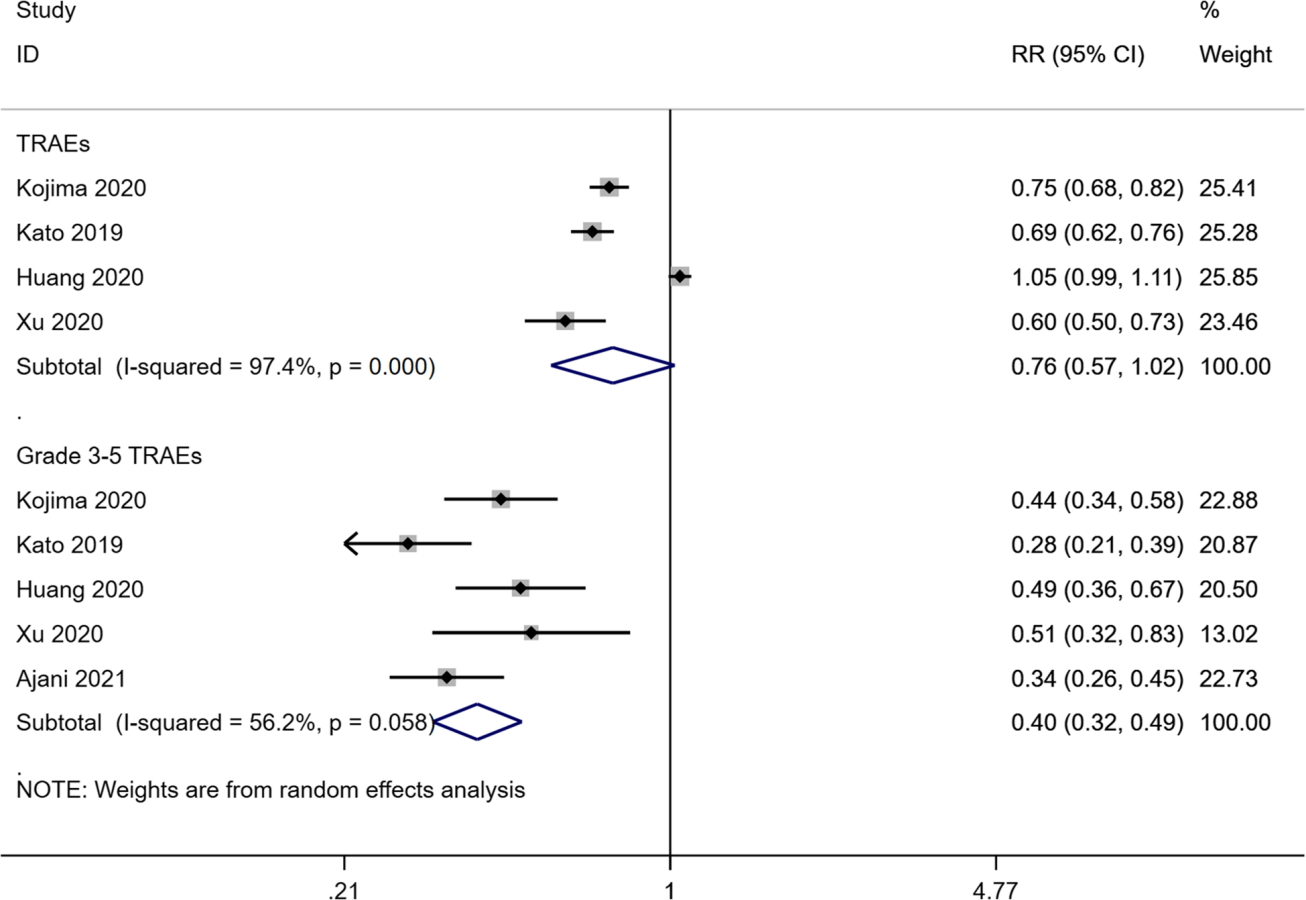
Forest plots of RR for TRAEs in patients treated with PD-1 inhibitors versus chemotherapy. Abbreviations. RR: relative risk; TRAEs: treatment-related adverse events

Any grade TRAEs data were available from four studies [15–18]. The results showed that there was no significant difference in the incidence of any grade TRAEs between the two groups (RR: 0.76, 95% CI: 0.57–1.02, *P* = 0.070; heterogeneity: I^2^ = 97.4%, *P* < 0.001) (Fig. 3). In the stratification of TRAEs, grade 3–5 TRAE data from all the five studies [15–19] showed that the PD-1 inhibitor group had significantly reduced risk of the incidence of grade 3–5 TRAEs compared with the chemotherapy group (RR: 0.40, 95% CI: 0.32–0.49, *P* < 0.001; heterogeneity: I^2^ = 56.2%, *P* = 0.058) (Fig. 3).

### OS and PD-L1 expression status

**Fig. 4:**
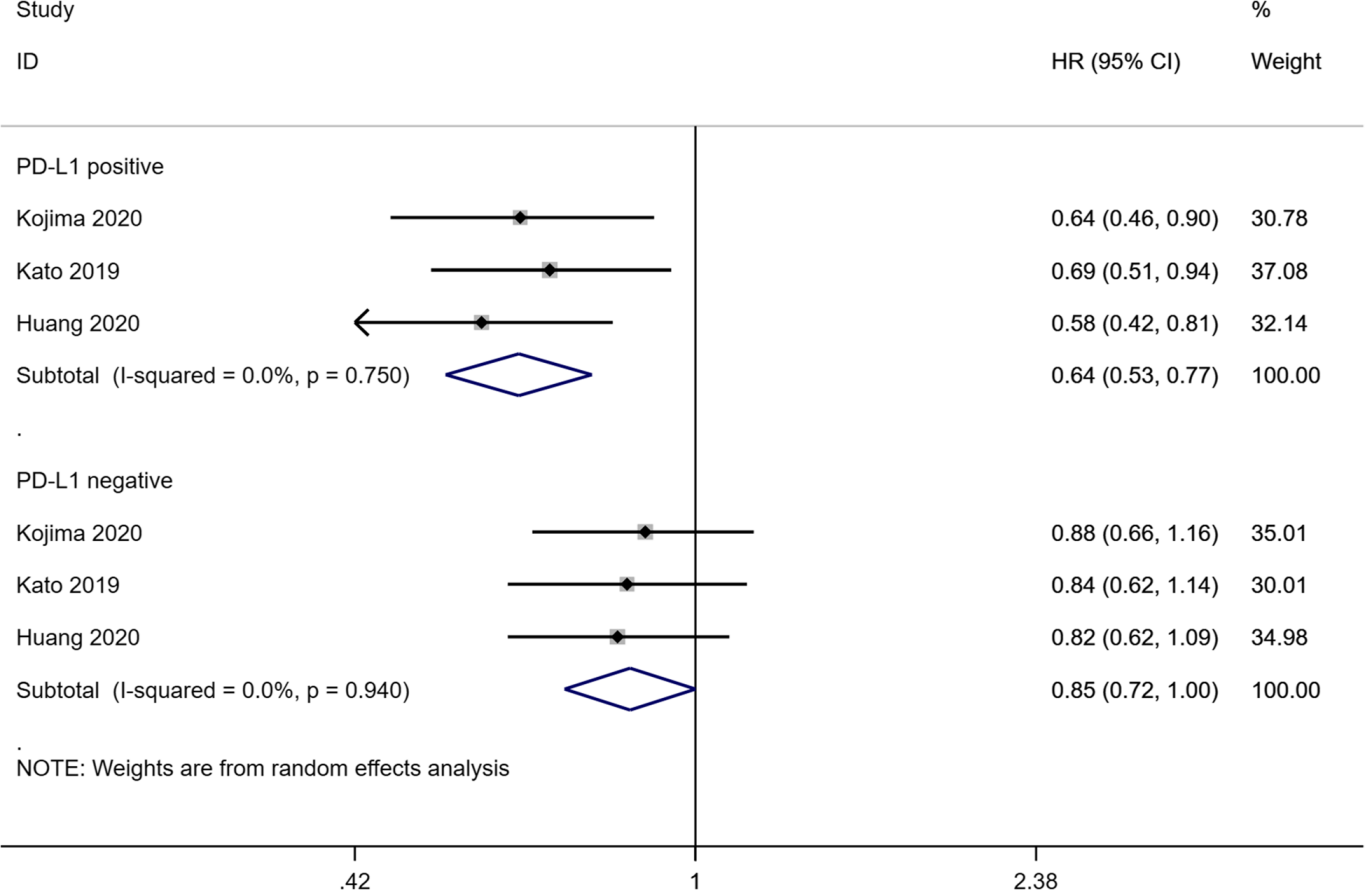
Forest plots of HR for OS in the patients with either positive or negative PD-L1 expression assigned to the PD-1 inhibitor group, compared with those in the chemotherapy group. Abbreviations. HR: hazard ratio; OS: overall survival

Three studies [15–17] analyzed OS in PD-L1 positive patients, which included 636 patients in the PD-1 inhibitor group and 632 patients in the chemotherapy group, showing that PD-1 inhibitor monotherapy significantly lowered the mortality risk in PD-L1 positive patients compared with chemotherapy (HR: 0.64, 95% CI: 0.53–0.77, *P* < 0.001; heterogeneity: I^2^ = 0.0%, *P* = 0.750) (Fig. 4). The OS data in PD-L1 negative patients were also available in the above study, including 636 patients in the PD-1 inhibitor group and 632 patients in the chemotherapy group. OS did was not significantly different between PD-L1 negative patients who were treated with PD-1 inhibitors as a monotherapy and those treated with chemotherapy (HR: 0.85, 95% CI: 0.72–1.00, *P* = 0.050; heterogeneity: I^2^ = 0.0%, *P* = 0.940) (Fig. 4). Notably, there was a significant difference in the OS with PD-1 inhibitors in patients with positive and negative PD-L1 expression compared with chemotherapy (*P* = 0.026 for interaction).

### Sensitivity analyses and publication bias

Sensitivity analyses showed that the combined results of OS were steady once the included studies were removed individually. The results are shown in Table 4. Because less than 10 studies were included, publication bias could not be assessed.

**Table 4:** The heterogeneity of meta-analysis after the following studies were removed

Study	I^**2**^ (%)	P_**heterogeneity**_	HR (95%)
Kojima 2020, KEYNOTE-181	0.0	0.923	0.72 (0.64, 0.81)
Kato 2019, ATTRACTION-3	0.0	0.882	0.72 (0.65, 0.81)
Huang 2020, ESCORT	0.0	0.856	0.74 (0.66, 0.83)
Xu 2020, ORIENT-2	0.0	0.848	0.74 (0.66, 0.82)
Ajani 2021, RATIONALE 302	0.0	0.895	0.75 (0.66, 0.84)

## Discussion

As one of the most important immune checkpoint protein, PD-1 expresses on activated T cells, B cells, natural killer cells, monocytes, and dendritic cells [20, 21], and plays a critical role in inhibiting immune responses and promoting self-tolerance by suppressing the activity of T cells; therefore, anti-PD-1 therapies have significantly evolved for cancer treatment in the era of immunotherapy [22–24]. We conducted this meta-analysis on all published RCTs data to systematically evaluate the therapeutic efficacy and safety of approved anti-PD-1 medications in patients with advanced ESCC refractory or intolerant to first-line regimens.

In the published data from five multicenter RCTs with 1970 advanced ESCC patients, our pooled analysis revealed that PD-1 inhibitors as second-line therapy resulted in better OS and ORR in advanced ESCC patients than chemotherapy, especially significantly prolonged OS in patients with positive PD-L1 status, and were associated with a lower incidence of grade 3–5 TRAEs. However, no statistical difference was obtained between PD-1 inhibitor and chemotherapy groups for PFS, DCR, and incidence of any grade TRAEs. Moreover, PD-1 inhibitors did not significantly prolong OS in patients with negative PD-L1 status.

In our meta-analysis, second-line PD-1 blockade decreased the risk of death by 27% in patients with advanced ESCC when comparing conventional chemotherapy (HR = 0.73, *P* < 0.001). The OS results of the present study was consistent with those reported in previous RCTs [16–19], except the KEYNOTE-181 study (pembrolizumab) [15], which did not meet the primary endpoint of OS in patients with ESCC. To date, published trials have reported varying ORR data. In the KEYNOTE-181 study (pembrolizumab), ESCORT study (camrelizumab), and RATIONALE study (tislelizumab), ORR in the PD-1 inhibitor group was significantly higher than that in the chemotherapy group [15, 17, 19], but similar to that in chemotherapy group in the ATTRACTION-3 study (nivolumab) and ORIENT-2 study (sintilimab) [16, 18]. Our study showed that patients with PD-1 blockade had an objective response of 1.89 times higher than patients treated with chemotherapy.

PFS and DCR were not improved with second-line PD-1 inhibitors versus chemotherapy in the present study, and a similar result has been found in some researches on other types of cancers [25–27]. A possible explanation was that treatment effects of immunotherapy might need more time to become apparent, but became more durable compared with chemotherapy [28].

PD-1 inhibitors have evolved for advanced cancer treatment; however, the favorable efficacy of the inhibitors has not been observed in the overall population [29]. In recent years, numerous studies have explored potentially applicable biomarkers for selecting suitable patients to receive immunotherapy, especially PD-L1 expression status [30, 31]. KEYNOTE-590 have shown that advanced EC patients with low PD-L1 expression experienced an obviously modest survival benefit than that with high PD-L1 expression treated with pembrolizumab plus chemotherapy compared with placebo plus chemotherapy [32]. However, KEYNOTE-181study, ATTRACTION-3 study, ESCORT study and a meta-analysis [33] of eight RCTs revealed that PD-L1 expression level was not a determinant of the OS benefit. Accordingly, the predictive and prognostic roles of PD-L1 expression status remain controversial. Our study confirmed that PD-L1 expression status could certainly be a biomarker for selecting more suitable patients to receive PD-1 blockade therapy, which was in accordance with the Food and Drug Administration’s approval of second-line pembrolizumab monotherapy for recurrent, locally advanced or metastatic ESCC patients expressing PD-L1 in the United States. Considering the high cost of anti-PD-1/PD-L1 medications, the optimal biomarkers that can predict the efficacy of PD-1/PD-L1 inhibitors should be the next priority for benefiting more general patients [34].

Among the five PD-1 inhibitors used in trials that were included in our meta-analysis, camrelizumab led to the highest incidence of TRAEs (89%), and sintilimab led to the lowest inicidence (54.5%) [15–17, 19]. The most common TRAEs of pembrolizumab, nivolumab, and camrelizumab were fatigue (11.8%), rash (11%), and reactive cutaneous capillary endothelial proliferation (79.8%), respectively [15–17]. Meanwhile, asthenia, fatigue, decreased appetite were also common TRAEs with those three drugs. Hypothyroidism was the second common TRAE in the pembrolizumab and camrelizumab groups, but was not common in the nivolumab group [15–17]. Gastrointestinal events such as nausea, vomiting and diarrhea were more observed with pembrolizumab and nivolumab than with camrelizumab, and hematologic issues were more common with camrelizumab [15–17]. However, the overall incidence of grade 3–5 TRAEs of the five drugs were similar, ranging from 19 to 22.2%, and anemia was ranked as the most common grade 3–5 TRAEs [15–19]. Moreover, TRAEs led to death in 1.6% patients in the pembrolizumab group, 5.2% in the nivolumab group, 3.1% in the camrelizumab group, and 14% in the tislelizumab group [15–17, 19]. In the present study, while there was no statistical difference in the incidence of any grade TRAEs between the two groups, the incidence of TRAEs of grades 3 or worse in the PD-1 inhibitors group was lower than that in the chemotherapy group. Therefore, our present study showed that PD-1 inhibitors have a favorable safety profile, consistent with previous studies in other tumour types [35, 36].

Since improvement in the activity of the immune system, PD-1 blockade has some side effects related to local or systemic inflammatory reactions, which are commonly coined immune-related adverse events (irAEs) of PD-1 blockade [37, 38]. The mechanisms underlying the development of irAEs are thought to be the enhancement of T-cell activation and proliferation, humoral autoimmunity boosting, an increase in the level of cytokines, and abrogation of regulatory T-cell functions [38]. The irAEs commonly affect the gastrointestinal tract, endocrine glands, skin, liver, and lung [37], which impaired the quality of life of patients to some extent. In total, 89% patients in the camrelizumab group of the ESCORT study developed irAEs, including reactive capillary endothelial proliferation (80%), hypothyroidism (19%), skin reaction (9%), hepatitis (8%), pneumonitis (7%), and hyperthyroidism (6%). However, in comparison, a fewer incidence of irAEs was reported in the pembrolizumab group of KEYNOTE-181 study (23.2%), with hypothyroidism (11.5%) as the most common one, pneumonitis (4.8%) and hyperthyroidism (4.1%) as the second and third. Since anti-PD-1 treatment is a new management for malignancy, its AEs, particularly irAEs, should be further investigated.

This study had some limitations. First, the number of retrieved studies was relatively small as only five open-label RCTs were selected with a limited number of patients. Second, PD-L1 expression was quantified with CPS in the KEYNOTE-181 study and RATIONALE 302 study, while the other three studies used TPS to assess the status of PD-L1 expression, thereby showing significant heterogeneity among the selected studies. Third, two included studies were still not published in extenso, which may have also increased heterogeneity of this meta.

## Conclusions

In conclusion, this study is the first meta-analysis to systematically review the clinical efficacy and therapeutic safety of PD-1 inhibitors in patients with advanced ESCC in second-line setting. PD-1 inhibitors possessed better overall survival and safety compared chemotherapy in advanced ESCC. PD-1 inhibitor monotherapy as a second-line treatment for advanced ESCC patients should be confirmed.

## Data Availability

The datasets generated and analyzed during the current study are available from the corresponding author on reasonable request.

## Abbreviations

PD-1: programmed cell death protein 1
ESCC: esophageal squamous cell carcinoma
ASCO: American Society of Clinical Oncology
ESMO: European Society for Medical Oncology
OS: overall survival.
PFS: progression-free survival.
ORR: objective response rate.
DCR: disease control rate.
TRAE: streatment-related adverse events.
HR: hazard ratio.
CI: confidence interval.
PD-L1: programmed death-ligand 1.
RR: relative risk.
EC: esophageal cancer.
RCT: srandomized controlled trials.
TPS: tumor proportion score.
CPS: combined positive score.
IrAE: simmune-related adverse events.

## References

[1] Bray F, Ferlay J, Soerjomataram I, Siegel RL, Torre LA, Jemal A (2018). Global cancer statistics 2018: GLOBOCAN estimates of incidence and mortality worldwide for 36 cancers in 185 countries. CA Cancer J Clin.

[2] Fitzmaurice C, Abate D, Abbasi N, Abbastabar H, Abd-Allah F, Abdel-Rahman O (2019). Global, regional, and National Cancer Incidence, mortality, years of life lost, years lived with disability, and disability-adjusted life-years for 29 Cancer groups, 1990 to 2017: a systematic analysis for the global burden of disease study. JAMA ONCOL.

[3] Ter Veer E, Haj MN, van Valkenhoef G, Ngai LL, Mali R, Anderegg MC (2016). The efficacy and safety of first-line chemotherapy in advanced Esophagogastric Cancer: a network Meta-analysis. J Natl Cancer Inst.

[4] Dutton SJ, Ferry DR, Blazeby JM, Abbas H, Dahle-Smith A, Mansoor W, Thompson J, Harrison M, Chatterjee A, Falk S, Garcia-Alonso A, Fyfe DW, Hubner RA, Gamble T, Peachey L, Davoudianfar M, Pearson SR, Julier P, Jankowski J, Kerr R, Petty RD (2014). Gefitinib for oesophageal cancer progressing after chemotherapy (COG): a phase 3, multicentre, double-blind, placebo-controlled randomised trial. LANCET ONCOL.

[5] Hecht JR, Bang YJ, Qin SK, Chung HC, Xu JM, Park JO, Jeziorski K, Shparyk Y, Hoff PM, Sobrero A, Salman P, Li J, Protsenko SA, Wainberg ZA, Buyse M, Afenjar K, Houé V, Garcia A, Kaneko T, Huang Y, Khan-Wasti S, Santillana S, Press MF, Slamon D (2016). Lapatinib in combination with Capecitabine plus Oxaliplatin in human epidermal growth factor receptor 2-positive advanced or metastatic gastric, esophageal, or gastroesophageal adenocarcinoma: TRIO-013/LOGiC--A randomized phase III trial. J Clin Oncol.

[6] Suntharalingam M, Winter K, Ilson D, Dicker AP, Kachnic L, Konski A, Chakravarthy AB, Anker CJ, Thakrar H, Horiba N, Dubey A, Greenberger JS, Raben A, Giguere J, Roof K, Videtic G, Pollock J, Safran H, Crane CH (2017). Effect of the addition of Cetuximab to paclitaxel, cisplatin, and radiation therapy for patients with esophageal Cancer: the NRG oncology RTOG 0436 phase 3 randomized clinical trial. JAMA ONCOL.

[7] Waddell T, Chau I, Cunningham D, Gonzalez D, Okines AFC, Okines C, Wotherspoon A, Saffery C, Middleton G, Wadsley J, Ferry D, Mansoor W, Crosby T, Coxon F, Smith D, Waters J, Iveson T, Falk S, Slater S, Peckitt C, Barbachano Y (2013). Epirubicin, oxaliplatin, and capecitabine with or without panitumumab for patients with previously untreated advanced oesophagogastric cancer (REAL3): a randomised, open-label phase 3 trial. The Lancet Oncology.

[8] Muro K, Lordick F, Tsushima T, Pentheroudakis G, Baba E, Lu Z, et al. Pan-Asian adapted ESMO clinical practice guidelines for the management of patients with metastatic oesophageal cancer: a JSMO-ESMO initiative endorsed by CSCO, KSMO, MOS. SSO and TOS *ANN ONCOL*. 2019;30(1):34–43. 10.1093/annonc/mdy498. 10.1093/annonc/mdy498 30475943

[9] Shim HJ, Cho SH, Hwang JE, Bae WK, Song SY, Cho SB, Lee WS, Joo YE, Na KJ, Chung IJ (2010). Phase II study of docetaxel and cisplatin chemotherapy in 5-fluorouracil/cisplatin pretreated esophageal cancer. Am J Clin Oncol.

[10] Rong L, Liu Y, Hui Z, Zhao Z, Zhang Y, Wang B, Yuan Y, Li W, Guo L, Ying J, Song Y, Wang L, Zhou Z, Xue L, Lu N (2019). PD-L1 expression and its clinicopathological correlation in advanced esophageal squamous cell carcinoma in a Chinese population. Diagn Pathol.

[11] Doi T, Piha-Paul SA, Jalal SI, Mai-Dang H, Saraf S, Koshiji M (2016). Updated results for the advanced esophageal carcinoma cohort of the phase Ib KEYNOTE-028 study of pembrolizumab (MK-3475). J CLIN ONCOL.

[12] Kudo T, Hamamoto Y, Kato K, Ura T, Kojima T, Tsushima T, Hironaka S, Hara H, Satoh T, Iwasa S, Muro K, Yasui H, Minashi K, Yamaguchi K, Ohtsu A, Doki Y, Kitagawa Y (2017). Nivolumab treatment for oesophageal squamous-cell carcinoma: an open-label, multicentre, phase 2 trial. The Lancet Oncology.

[13] Shah MA, Kojima T, Hochhauser D, Enzinger P, Raimbourg J, Hollebecque A*, Lordick F, Kim SB, Tajika M, Kim HT, Lockhart AC, Arkenau HT, el-Hajbi F, Gupta M, Pfeiffer P, Liu Q, Lunceford J, Kang SP, Bhagia P, Kato K* Efficacy and Safety of Pembrolizumab for Heavily Pretreated Patients With Advanced,Metastatic Adenocarcinoma or Squamous Cell Carcinoma of the Esophagus: The Phase 2 KEYNOTE-180 Study. JAMA ONCOL 2019; 5(4):546–550, Efficacy and Safety of Pembrolizumab for Heavily Pretreated Patients With Advanced, Metastatic Adenocarcinoma or Squamous Cell Carcinoma of the Esophagus, DOI: 10.1001/jamaoncol.2018.5441. 10.1001/jamaoncol.2018.5441 PMC6459121 30570649

[14] Higgins JP, Altman DG, Gøtzsche PC, Jüni P, Moher D, Oxman AD (2011). The Cochrane Collaboration's tool for assessing risk of bias in randomised trials. BMJ.

[15] Kojima T, Shah MA, Muro K, Francois E, Adenis A, Hsu CH, Doi T, Moriwaki T, Kim SB, Lee SH, Bennouna J, Kato K, Shen L, Enzinger P, Qin SK, Ferreira P, Chen J, Girotto G, de la Fouchardiere C, Senellart H, al-Rajabi R, Lordick F, Wang R, Suryawanshi S, Bhagia P, Kang SP, Metges JP, on behalf of the KEYNOTE-181 Investigators (2020). Randomized phase III KEYNOTE-181 study of Pembrolizumab versus chemotherapy in advanced esophageal Cancer. J Clin Oncol.

[16] Kato K, Cho BC, Takahashi M, Okada M, Lin C, Chin K (2019). Nivolumab versus chemotherapy in patients with advanced oesophageal squamous cell carcinoma refractory or intolerant to previous chemotherapy (ATTRACTION-3): a multicentre, randomised, open-label, phase 3 trial. The Lancet Oncology.

[17] Huang J, Xu J, Chen Y, Zhuang W, Zhang Y, Chen Z*, Chen J., Zhang H., Niu Z., Fan Q., Lin L., Gu K., Liu Y., Ba Y., Miao Z., Jiang X., Zeng M., Chen J., Fu Z., Gan L., Wang J., Zhan X., Liu T., Li Z., Shen L., Shu Y., Zhang T., Yang Q., Zou J., Luo S., Peng F., Wu G., Xu N., Zhao L., Ma D., Qin S., Ren W., Li E., Lu H., Pan Y., Xiong J., Yuan Y., Bai Y., Chen L., Hu Y., Zhang L., Gao Y.* Camrelizumab versus investigator's choice of chemotherapy as second-line therapy for advanced or metastatic oesophageal squamous cell carcinoma (ESCORT): a multicentre,randomised, open-label, phase 3 study. LANCET ONCOL 2020; 21(6):832–842, Camrelizumab versus investigator's choice of chemotherapy as second-line therapy for advanced or metastatic oesophageal squamous cell carcinoma (ESCORT): a multicentre, randomised, open-label, phase 3 study, DOI: 10.1016/S1470-2045(20)30110-8. 10.1016/S1470-2045(20)30110-8 32416073

[18] Xu J, Li Y, Fan Q, Shu Y, Wang Y (2020). Sintilimab in patients with advanced esophageal squamous cell carcinoma refractory to previous chemotherapy: A randomized, open-label phase II trial (ORIENT-2). J CLIN ONCOL.

[19] Ajani J, El Hajbi F, Cunningham D, Alsina M, Thuss-Patience P, Scagliotti G (2021). O-15 randomized, phase 3 study of second-line tislelizumab vs chemotherapy in advanced or metastatic esophageal squamous cell carcinoma (RATIONALE 302) in the overall population and Europe/North America subgroup. Ann Oncol.

[20] He J, Hu Y, Hu M, Li B (2015). Development of PD-1/PD-L1 pathway in tumor immune microenvironment and treatment for non-small cell lung Cancer. Sci Rep.

[21] Pedoeem A, Azoulay-Alfaguter I, Strazza M, Silverman GJ, Mor A (2014). Programmed death-1 pathway in cancer and autoimmunity. Clin Immunol.

[22] Han Y, Liu D, Li L (2020). PD-1/PD-L1 pathway: current researches in cancer. Am J Cancer Res.

[23] Salmaninejad A, Valilou SF, Shabgah AG, Aslani S, Alimardani M, Pasdar A, Sahebkar A (2019). PD-1/PD-L1 pathway: basic biology and role in cancer immunotherapy. J Cell Physiol.

[24] Salmaninejad A, Khoramshahi V, Azani A, Soltaninejad E, Aslani S, Zamani MR, Zal M, Nesaei A, Hosseini SM (2018). PD-1 and cancer: molecular mechanisms and polymorphisms. IMMUNOGENETICS.

[25] Fradet Y, Bellmunt J, Vaughn DJ, Lee JL, Fong L, Vogelzang NJ, Climent MA, Petrylak DP, Choueiri TK, Necchi A, Gerritsen W, Gurney H, Quinn DI, Culine S, Sternberg CN, Nam K, Frenkl TL, Perini RF, de Wit R, Bajorin DF (2019). Randomized phase III KEYNOTE-045 trial of pembrolizumab versus paclitaxel, docetaxel, or vinflunine in recurrent advanced urothelial cancer: results of >2 years of follow-up. Ann Oncol.

[26] Borghaei H, Paz-Ares L, Horn L, Spigel DR, Steins M, Ready NE, Chow LQ, Vokes EE, Felip E, Holgado E, Barlesi F, Kohlhäufl M, Arrieta O, Burgio MA, Fayette J, Lena H, Poddubskaya E, Gerber DE, Gettinger SN, Rudin CM, Rizvi N, Crinò L, Blumenschein GR, Antonia SJ, Dorange C, Harbison CT, Graf Finckenstein F, Brahmer JR (2015). Nivolumab versus docetaxel in advanced nonsquamous non-small-cell lung Cancer. N Engl J Med.

[27] Liang H, Liu Z, Cai X, Pan Z, Chen D, Li C, Chen Y, He J, Liang W (2019). PD-(L)1 inhibitors vs. chemotherapy vs. their combination in front-line treatment for NSCLC: an indirect comparison. Int J Cancer.

[28] Borcoman E, Nandikolla A, Long G, Goel S, Le Tourneau C (2018). Patterns of response and progression to immunotherapy. Am Soc Clin Oncol Educ Book.

[29] Cha JH, Chan LC, Li CW, Hsu JL, Hung MC (2019). Mechanisms controlling PD-L1 expression in Cancer. Mol Cell.

[30] Zang YS, Dai C, Xu X, Cai X, Wang G, Wei J, Wu A, Sun W, Jiao S, Xu Q (2019). Comprehensive analysis of potential immunotherapy genomic biomarkers in 1000 Chinese patients with cancer. Cancer Med.

[31] Greally M, Chou JF, Chatila WK, Margolis M, Capanu M, Hechtman JF, Tuvy Y, Kundra R, Daian F, Ladanyi M, Kelsen DP, Ilson DH, Berger MF, Tang LH, Solit DB, Diaz LA, Schultz N, Janjigian YY, Ku GY (2019). Clinical and molecular predictors of response to immune checkpoint inhibitors in patients with advanced Esophagogastric Cancer. Clin Cancer Res.

[32] Sun JM, Shen L, Shah MA, Enzinger P, Adenis A, Doi T, Kojima T, Metges JP, Li Z, Kim SB, Cho BC, Mansoor W, Li SH, Sunpaweravong P, Maqueda MA, Goekkurt E, Hara H, Antunes L, Fountzilas C, Tsuji A, Oliden VC, Liu Q, Shah S, Bhagia P, Kato K (2021). Pembrolizumab plus chemotherapy versus chemotherapy alone for first-line treatment of advanced oesophageal cancer (KEYNOTE-590): a randomised, placebo-controlled, phase 3 study. LANCET.

[33] Shen X, Zhao B. Efficacy of PD-1 or PD-L1 inhibitors and PD-L1 expression status in cancer: meta-analysis. BMJ. 2018;362:k3529. 10.1136/bmj.k3529. 10.1136/bmj.k3529 PMC6129950 30201790

[34] Baba Y, Nomoto D, Okadome K, Ishimoto T, Iwatsuki M, Miyamoto Y, et al. Tumor immune microenvironment and immune checkpoint inhibitors in esophageal squamous cell carcinoma. Cancer Sci. 2020;111(9):3132–41. 10.1111/cas.14541. 10.1111/cas.14541 PMC7469863 32579769

[35] Even C, Wang HM, Li SH, Ngan RK, Dechaphunkul A, Zhang L, et al. Phase II, Randomized Study of Spartalizumab (PDR001), an Anti–PD-1 Antibody, versus Chemotherapy in Patients with Recurrent/Metastatic Nasopharyngeal Cancer. In: Phase II, randomized study of Spartalizumab (PDR001), an anti-PD-1 antibody. CLIN CANCER RES: Versus Chemotherapy in Patients With Recurrent/Metastatic Nasopharyngeal Cancer; 2021. 10.1158/1078-0432.CCR-21-0822 34433653

[36] Shi Y, Duan J, Guan Q, Xue P, Zheng Y. Effectivity and safety of PD-1/PD-L1 inhibitors for different level of PD-L1-positive, advanced NSCLC: a meta-analysis of 4939 patients from randomized controlled trials. Int Immunopharmacol. 2020;84:106452. 10.1016/j.intimp.2020.106452. 10.1016/j.intimp.2020.106452 32339922

[37] Nishijima TF, Shachar SS, Nyrop KA, Muss HB. Safety and tolerability of PD-1/PD-L1 inhibitors compared with chemotherapy in patients with advanced Cancer: a Meta-analysis. Oncologist. 2017;22(4):2016–419. 10.1634/theoncologist.2016-0419. 10.1634/theoncologist.2016-0419 PMC5388381 28275115

[38] Postow MA, Sidlow R, Hellmann MD. Immune-related adverse events associated with immune checkpoint blockade. N Engl J Med. 2018;378(2):158–68. 10.1056/NEJMra1703481. 10.1056/NEJMra1703481 29320654

